# Pedunculopontine Nucleus Dysconnectivity Correlates With Gait Impairment in Parkinson’s Disease: An Exploratory Study

**DOI:** 10.3389/fnagi.2022.874692

**Published:** 2022-07-08

**Authors:** Stephen Joza, Richard Camicioli, W. R. Wayne Martin, Marguerite Wieler, Myrlene Gee, Fang Ba

**Affiliations:** ^1^Division of Neurology, Department of Medicine, University of Alberta, Edmonton, AB, Canada; ^2^Department of Medicine, University of Alberta, Edmonton, AB, Canada; ^3^Department of Physical Therapy, Faculty of Rehabilitation Medicine, University of Alberta, Edmonton, AB, Canada

**Keywords:** Parkinson’s disease, pedunculopontine nucleus, gait impairments, MRI, diffusion tensor imaging

## Abstract

**Background:**

Gait impairment is a debilitating and progressive feature of Parkinson’s disease (PD). Increasing evidence suggests that gait control is partly mediated by cholinergic signaling from the pedunculopontine nucleus (PPN).

**Objective:**

We investigated whether PPN structural connectivity correlated with quantitative gait measures in PD.

**Methods:**

Twenty PD patients and 15 controls underwent diffusion tensor imaging to quantify structural connectivity of the PPN. Whole brain analysis using tract-based spatial statistics and probabilistic tractography were performed using the PPN as a seed region of interest for cortical and subcortical target structures. Gait metrics were recorded in subjects’ medication ON and OFF states, and were used to determine if specific features of gait dysfunction in PD were related to PPN structural connectivity.

**Results:**

Tract-based spatial statistics revealed reduced structural connectivity involving the corpus callosum and right superior corona radiata, but did not correlate with gait measures. Abnormalities in PPN structural connectivity in PD were lateralized to the right hemisphere, with pathways involving the right caudate nucleus, amygdala, pre-supplementary motor area, and primary somatosensory cortex. Altered connectivity of the right PPN-caudate nucleus was associated with worsened cadence, stride time, and velocity while in the ON state; altered connectivity of the right PPN-amygdala was associated with reduced stride length in the OFF state.

**Conclusion:**

Our exploratory analysis detects a potential correlation between gait dysfunction in PD and a characteristic pattern of connectivity deficits in the PPN network involving the right caudate nucleus and amygdala, which may be investigated in future larger studies.

## Introduction

Gait impairment is a common, debilitating, and progressive feature of Parkinson’s disease (PD) (Mirelman et al., 2019). Patients with gait impairment are known to have greater fall risk (Creaby and Cole, 2018), cognitive decline, faster disease progression (Arie et al., 2017), and earlier mortality (Bäckström et al., 2018). In contrast to the other cardinal features of PD, gait disorders are frequently refractory to dopamine-replacement medications, thus implicating the involvement of additional non-dopaminergic pathways.

Changes in cholinergic, rather than dopaminergic, neurotransmission have been implicated in gait/balance disturbance and falls associated with PD (Mancini et al., 2015; Henderson et al., 2016; Perez-Lloret et al., 2016; Janickova et al., 2017; Wilson et al., 2021). Cholinergic-mediated cortical and subcortical processing involving the pedunculopontine nucleus (PPN) appears to be critical for normal gait performance. Anatomically, the PPN has been shown to have connections with numerous areas including the vestibular nuclei (Aravamuthan and Angelaki, 2012), deep cerebellar nuclei (Hazrati and Parent, 1992), premotor, supplementary motor, and primary motor cortices (Aravamuthan and Angelaki, 2012), thalamocortical pathways (Steriade et al., 1990), basal ganglia nuclei (Charara et al., 1996), and the limbic system (Xi et al., 2012). Functionally, extracellular recordings show increased single unit activity in the PPN during imagined gait, suggesting its importance in motor planning and gait initiation (Lau et al., 2015). Selective cholinergic lesions of the PPN in experimental models can induce freezing of gait (FOG) and postural instability without affecting dopamine-responsive motor symptoms (Grabli et al., 2013; Chambers et al., 2021). PPN neuronal loss is evident in PD (Rinne et al., 2008) and alterations in PPN microstructural integrity are associated with gait impairment and postural instability and may predict the future development of these problems in PD patients (Craig et al., 2020). Moreover, alterations in PPN structural connectivity are associated with FOG and attentional control during dual-task gait (Fling et al., 2013, 2014; Mancini et al., 2015).

Diffusion tensor imaging (Eidelberg et al., 1990) uses quantitative metrics to describe microstructural changes in neurodegenerative diseases (Table 1; Alexander et al., 2011). Several studies using DTI in PD have examined correlations with clinical measures, such as the Unified Parkinson’s Disease Rating Scale (UPDRS) (Vercruysse et al., 2015; Li et al., 2018), and Timed Up and Go (TUG) (Surova et al., 2016). However, the anatomic bases for quantitative gait measures, which can be grouped into distinct subdomains, for example, pace and rhythm (Pieruccini-Faria et al., 2021), have not been well studied in PD. Moreover, the correlates of gait in the ON and OFF state in relation to anatomic connectivity to the PPN have not been extensively examined. In this study, we used DTI-derived tract-based spatial statistics (TBSS) and tractography using the PPN as seed region of interest (ROI) to quantify structural connectivity in participants with PD relative to healthy controls. We next assessed gait parameters in both the ON and OFF states to provide a better understanding of the role of the PPN and its connectivity to locomotion and gait centers and their relationship to the dopaminergic response. Finally, we evaluated whether the integrity of PPN structural connectivity correlated with gait parameters. This study addresses an emerging topic, since such assessments in relation to clinical parameters can potentially serve as biomarkers to monitor disease progression, particularly in gait impairment in PD.

**TABLE 1:** List of diffusion metrics and proposed interpretations.

Diffusion metric	Proposed interpretation
FA	Decreases are suggestive of reduced microstructural integrity of white matter
MD	Increases are suggestive of cellular damage, including edema, and necrosis, regardless of brain tissue type (e.g., gray matter or white matter)
AD	Changes are suggestive of axonal injury
RD	Changes are suggestive of demyelination

*AD, axial diffusivity; FA, fractional anisotropy; MD, mean diffusivity; RD, radial diffusivity.*

## Materials and Methods

### Participants

Participants in this study were described previously (Surkont et al., 2021). PD patients receiving optimal treatment with dopaminergic medications but with persistent gait impairments were recruited from the Movement Disorders Program at the University of Alberta. A consecutive series of optimally treated PD patients from our Movement Disorders Program (which sees ∼450 PD patients/year) were first screened with the UPDRS to identify those with gait or balance changes (a score ≥1 on items 13, or 14 or 15 on the Activities of Daily Living subsection of the UPDRS or a score of ≥1 on items 29 or 30 on the Motor subsection). Twenty-one PD subjects and 15 healthy controls were enrolled from June 2010 to February 2013. Community volunteers without neurological disorders were recruited as healthy controls. One PD subject was removed from analysis due to poor imaging quality. All subjects with PD were assessed by movement disorders neurologists who confirmed the diagnosis of idiopathic PD based on the United Kingdom Parkinson’s Disease Society Brain Bank criteria (Hughes et al., 2002). PD patients were assessed with the following to characterize disease status and gait impairment: full UPDRS, Berg Balance Scale (BBS), Dynamic Gait Index (DGI), New Freezing of Gait Questionnaire (nFOGQ), and the TUG, all of which have been shown to be valid tools in PD assessment. Most PD participants experienced motor fluctuations; the clinical assessments were performed in the “defined OFF” with all PD medication on hold for a minimum of 12 h, and “ON” states with regular morning dose of Parkinson medications. Levodopa equivalent doses were calculated based on published dose conversion factors (Schade et al., 2020). Objective gait assessments of two gait domains were measured in PD and in healthy controls: velocity and stride length (pace) and cadence and stride time (Fleisher et al., 2007). These were quantified at a self-selected pace (SSP) and fast pace (FP) using a 14-foot long computerized GaitRite^®^ mat. The clinical tests were performed during OFF and ON states. Lateralization of motor symptoms was computed as the ratio between the sum of UPDRS-III right-sided motor scores versus left-sided motor scores in the OFF state, where a ratio greater than 1.0 shows greater right-sided involvement.

### Ethics Approval

The study was approved by the University of Alberta Health Research Ethics Board, and all participants provided written informed consent prior to participation.

### MRI Acquisition and Processing

DTI acquisition and quality control for this cohort was previously described (Surkont et al., 2021). Briefly, structural and diffusion tensor data for each subject were obtained on a Siemens Sonata 1.5T MRI within 1 month of enrollment. Data were acquired using MPRAGE with TR/TE/TI 2,120/4/300 ms, flip angle 15°, 1.5 mm slice thickness and diffusion weighted spin echo echoplanar sequences (SE EPI: TR/TE 5,600/88 ms, FOV 220 × 220 mm, 35 slices × 2 mm/slice; *b* = 1,000 s/mm^2^, modified to acquire 30 diffusion directions with the acquisition voxel size of 2 mm × 2 mm × 2.5 mm). After in-plane interpolation, the voxel size was 1 mm × 1 mm × 2.5 mm. There was one b0 volume acquired. Two scans were acquired in the study, and the b0 volumes were registered to each other to minimize movement artifacts before averaging and correcting for eddy current using standard diffusion image processing tools included in the FMRIB Software Library (FSL, version 6.0).

#### Tract-Based Spatial Statistics

Voxelwise statistical analysis using TBSS was performed to analyze DTI metrics across whole-brain without any *a priori* hypothesis (Smith et al., 2006). All subjects’ fractional anisotropy (FA) data were aligned into a common space using the FSL non-linear registration tool (FNIRT). Next, a mean FA image was created and thinned to create a mean FA skeleton which represented the centers of all tracts common to the group. The non-linear warps and skeleton projection were subsequently applied to all subjects to create mean medial diffusivity (MD), radial diffusivity (RD), and axial diffusivity (AD) maps. Finally, each subject’s aligned FA, MD, RD, and AD data were then projected onto the skeleton and the resulting data fed into voxelwise cross-subject statistics. For ease of visualization, clusters of reduced FA were algorithmically thickened around the skeleton in FSL (tbss_fill). Significant clusters were localized using the JHU ICBM-DTI-81 White Matter Labels Atlas (Mori et al., 2008), and mean FA values in the subjects were calculated from each identified tract. Since there was only partial overlap of clusters with the atlas-identified tracts (Supplementary Figure 1), 4-mm diameter spheres centered around the peak coordinates of each significant cluster were additionally created and mean values from each subjects’ FA maps contained within the spheres were calculated (Ji et al., 2015).

#### Probabilistic Tractography

Probabilistic tractography was run using the PPN as the seed ROI and 28 cortical and subcortical target ROIs known to have projections to and from the PPN (French and Muthusamy, 2018). The left and right PPN ROIs were previously identified based on post-mortem MRI and histology (Alho et al., 2017) and were registered to sixth generation MNI152 space using FNIRT. Cortical and subcortical ROIs were derived from previously described brain atlases: Human Motor Area Template (Mayka et al., 2006), Basal Ganglia Human Area Template (Prodoehl et al., 2008), and the Harvard-Oxford brain atlas (distributed within FSL).

The BEDPOSTX diffusion model was applied to create probabilistic distributions of the diffusion parameters in each voxel and model crossing fibers (Behrens et al., 2003). Probabilistic fiber tracking was initiated from each voxel within a binarized PPN ROI seed mask in each subject’s native diffusion space with 10,000 streamline samples from each voxel at step length 0.5 mm and curvature threshold of 0.2 in either an unrestricted fashion or to subcortical and cortical targets. An exclusion mask of the midline removed pathways that crossed into the contralateral hemisphere. For each subject, probabilistic connectivity distribution maps were thresholded to 5% (Fling et al., 2013), binarized, and used to extract total tract volume and mean FA, MD, RD, and AD values for each tract in each subject. Mean values for PD and control subjects were then calculated.

#### Along-the-Tract Analysis

Fractional anisotropy, MD, RD, and AD values as a function of distance from seed to target were derived from FSL tractography data using custom MATLAB code. Values were binned to either 30 or 60 tract location points between the PPN and subcortical or cortical target, respectively. Given the tendency of signal fall-off as tractography approaches the target, the most terminal 5 or 10 points were excluded from the analysis in all subcortical and cortical targets, respectively.

### Statistical Analyses

Statistical analyses were performed using SPSS (Version 27, IBM Corporation, Armonk, NY, United States).

Demographic and clinical characteristics in the control and PD group were analyzed with Student’s *t*-test or one-way analysis of variance (ANOVA) with Tukey HSD *post hoc* testing for quantitative variables, and Chi-squared test for qualitative variables. One-way analyses of covariance (ANCOVA) were used to compare tractography data between control and PD subjects using age and sex as covariates.

TBSS data underwent voxelwise analysis using permutation-based non-parametric testing in FSL (randomize). Two-group unpaired *t*-tests with age and sex as covariates were conducted with 5,000 permutations per analysis and threshold-free cluster enhancement (Smith and Nichols, 2009) to assess group differences between PD and controls.

The FA, MD, RD, and AD values along either 25 or 50 tract points between the PPN and subcortical or cortical targets, respectively, were compared using Student’s *t*-test to assess along-the-tract difference per group.

Pearson’s product-moment correlations were run to assess the relationship between tractography data of interest and clinical gait metrics after assessing for relationship linearity and normality of distribution, as determined by the Shapiro–Wilk test.

Tract-based spatial statistics clusters, tractography diffusion metrics, and along-the-tract analyses were subjected to the Benjamini–Hochberg method to control the false discovery rate (alpha at 0.05) to correct for multiple comparisons (Benjamini and Hochberg, 1995). Analyses are presented with uncorrected *p*-values, and those not surviving false discovery rate correction are noted in the text.

## Results

### Demographic and Clinical Data

Participant demographic and clinical data are shown in Table 2. Seventeen of 20 patients had some degree of FOG in this study that recruited PD participants with gait impairment. When classifying the PD subjects (Jankovic et al., 1990), 13 were of the postural instability/gait disorder phenotype, 3 were tremor-dominant, and 4 were of indeterminate phenotype. Gait parameters demonstrated significant differences in velocity and stride length when comparing PD subjects versus controls; trends toward improved velocity and stride length, although not statistically significant, were observed when comparing PD subjects when OFF versus ON. The lateralization of motor symptoms was evenly distributed between PD subjects.

**TABLE 2:** Demographic and clinical characteristics of participants.

	Controls	PD	
		OFF state	ON state
*n*	15	20	
Age	60.8 ± 6.9	63.93 ± 9.19	
Sex (% male)	66.7	70.0	
MoCA	27.5 ± 2.1	25.5 ± 2.3*	
LEDD (mg)	*n*/*a*	904.0 ± 539.0	
UPDRS III	*n*/*a*	33.8 ± 9.0	23.4 ± 9.6^†^
UPDRS total	*n*/*a*	51.1 ± 13.6	34.0 ± 13.2^†^
BBS	56.0 ± 0	51.5 ± 4.0**	53.1 ± 3.1*
TUG (s)	6.9 ± 1.1	10.8 ± 4.9**	8.9 ± 1.9
DGI	23.1 ± 1.1	17.5 ± 3.8**	18.7 ± 2.7**
Velocity SSP (cm/s)	138.7 ± 20.6	106.4 ± 19.7**	113.3 ± 19.4**
Cadence SSP (step/min)	117.0 ± 9.9	116.4 ± 7.9	114.1 ± 8.5
Stride time SSP (s)	1.03 ± 0.09	1.0 ± 0.1	1.1 ± 0.1
Stride length SSP (cm)	142.1 ± 15.6	110.3 ± 19.5**	113.4 ± 17.4**
Velocity FP (cm/s)	188.2 ± 20.3	144.7 ± 28.2**	162.9 ± 26.2*
Cadence FP (step/min)	142.7 ± 11.8	134.2 ± 12.0	138.2 ± 11.1
Stride time FP (s)	0.9 ± 0.1	0.9 ± 0.1	0.9 ± 0.1
Stride length FP (cm)	158.6 ± 15.3	129.1 ± 19.8**	141.3 ± 18.4*

Data are presented as mean (standard deviation). *p < 0.05, **p < 0.01 versus control group. ^†^p < 0.05 versus OFF state. PD, Parkinson’s disease; MoCA, Montreal Cognitive Assessment; UPDRS, Unified Parkinson’s Disease Rating Scale; LEDD, levodopa equivalent daily dose (mg); BBS, Berg Balance Scale; TUG, Timed Up and Go; DGI, Dynamic Gait Index; SSP, self-selected pace; FP, fast pace.

### Tract-Based Spatial Statistics Analysis

**FIGURE 1:**
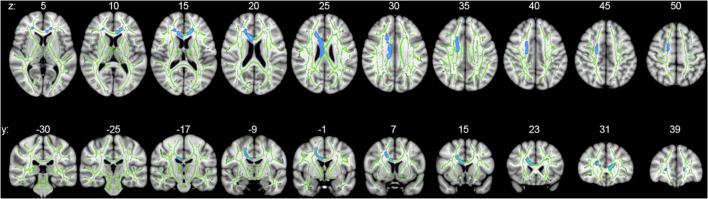
Whole-brain voxelwise statistical analysis using TBSS. Axial (upper series) and coronal (lower series) *p*-value statistical maps show clusters of significantly reduced FA in PD relative to control subjects in clusters coinciding with the body and genu of the corpus callosum and right superior corona radiata (blue: family-wise error-corrected, *p* < 0.05). For ease of visualization, clusters of reduced FA were algorithmically thickened around the skeleton. Results are superimposed on the mean tract fiber skeleton (green) and overlaid on the MNI152 1 mm T1 standard. Images are orientated by radiological convention (left hemisphere is on the right side).

Whole-brain voxelwise statistical analysis using TBSS identified clusters of decreased FA in PD subjects as compared to controls when adjusted for age and sex (Figure 1). Clusters of statistical significance coincided with the body (*p* = 0.039) and genu (*p* = 0.043) of the corpus callosum and the right superior corona radiata (*p* = 0.039; Table 3 and Figure 1). There were no areas of statistically significantly increased FA nor any differences in MD, RD, or AD values between PD and control subjects.

**TABLE 3:** Clusters identified by TBSS with significantly lower FA values in PD versus controls.

		MNI coordinates (mm)			
White matter tract location	Voxels	*x*	*y*	*z*	*p*-Value
Body of corpus callosum	762	7	14	22	**0.039**
Genu of corpus callosum	198	−15	35	9	**0.043**
Right superior corona radiata	389	19	−6	39	**0.039**

*MNI coordinates and p-values refer to coordinates of peak statistical significance in each cluster. Statistically significant correlations (p < 0.05) are bolded.*

Based on the identified clusters, mean FA values were extracted from the white matter tracts of interest and compared between PD and controls (Table 4). FA values were significantly reduced in both the body and genu of the corpus callosum, though not in the right superior corona radiata. Additionally, since each cluster had only partial overlap with each white matter tract (Supplementary Figure 1), the analysis was repeated by calculating the mean FA values contained within 4 mm spheres centered upon the coordinates of peak statistical significance within each cluster (Table 4). Significantly reduced mean FA values were observed in PD relative to control subjects in each of these areas.

**TABLE 4:** Comparison of mean FA values in white matter areas of interest identified by TBSS.

White matter area		FA		
		PD	Control	*p*-Value
Body of corpus callosum	Full tract	0.533 (0.029)	0.564 (0.037)	**0.010**
	Sphere	0.557 (0.039)	0.611 (0.058)	**0.002**
Genu of corpus callosum	Full tract	0.514 (0.029)	0.535 (0.033)	**0.038**
	Sphere	0.561 (0.028)	0.603 (0.045)	**0.005**
Right superior corona radiata	Full tract	0.435 (0.029)	0.444 (0.020)	0.333
	Sphere	0.399 (0.049)	0.462 (0.059)	**0.002**

*Statistically significant differences (p < 0.05) are bolded. Data are presented as mean (standard deviation).*

### Pedunculopontine Nucleus Probabilistic Tractography

**FIGURE 2:**
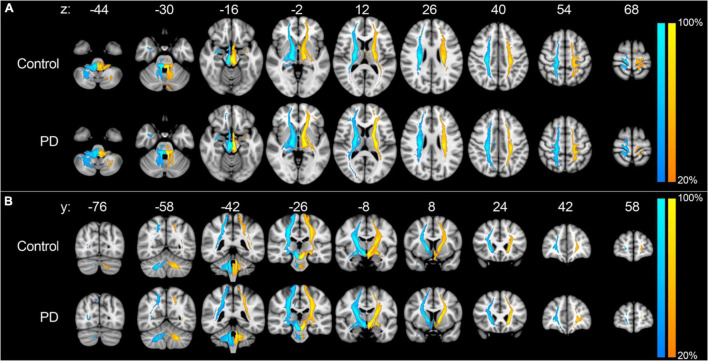
Unrestricted tractography using the PPN as seed region. Unrestricted connectivity maps using the right (blue-cyan) and left (orange-yellow) PPN as seed ROIs in the axial **(A)** and coronal **(B)** planes. Results are overlaid on the MNI152 1 mm T1 standard. Color bars reflect the percentage of subjects in which tracts overlapped, with warmer colors reflecting greater overlap. Images are orientated by radiological convention (left hemisphere is on the right side).

**FIGURE 3:**
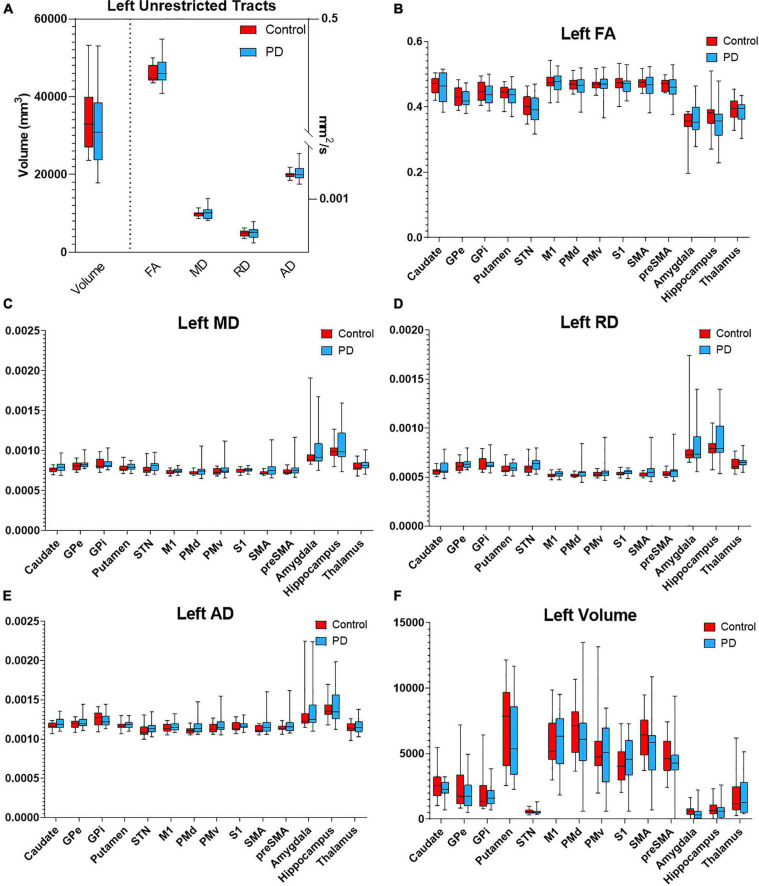
Diffusion metrics for PPN tractography in the left hemisphere. Box plots displaying diffusion metrics calculated from tractography using the left PPN as the seed ROI for unrestricted **(A)** and targeted tractography **(B–F)**. No significant differences were observed in FA **(B)**, MD **(C)**, RD **(D)**, AD **(E)**, and the tract volume **(F)**. AD, axial diffusivity; FA, fractional anisotropy; MD, mean diffusivity; RD, radial diffusivity; GPe, globus pallidus externa; GPi, globus pallidus interna; M1, primary motor cortex; PMd, dorsal premotor cortex; PMv, ventral premotor cortex; S1, primary sensory cortex; SMA, supplementary motor area; pre-SMA, pre- supplementary motor area; STN, subthalamic nucleus.

**FIGURE 4:**
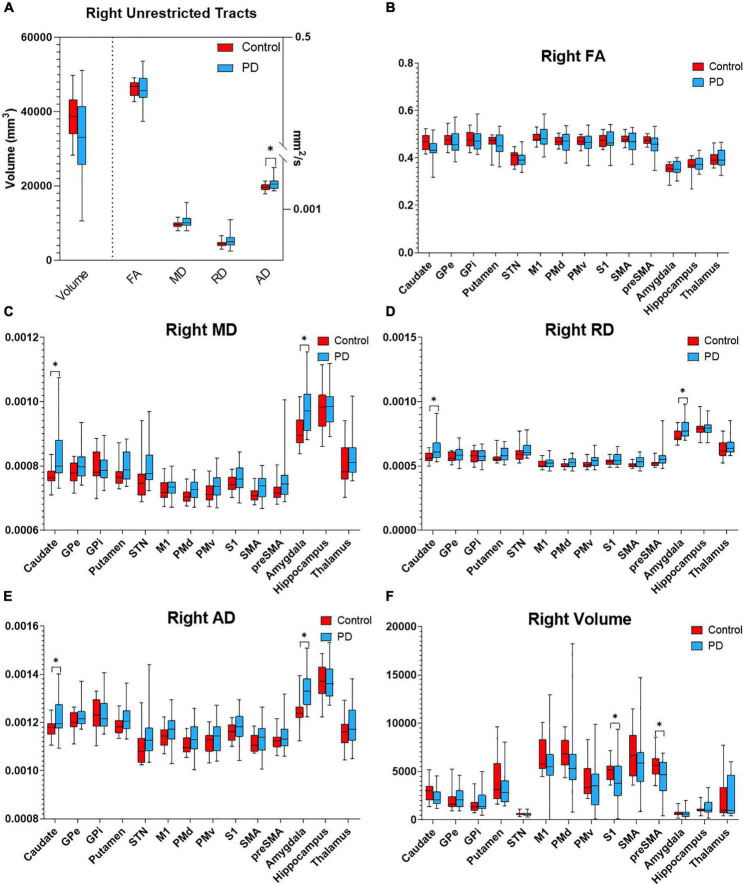
Diffusion metrics for PPN tractography in the right hemisphere. Box plots displaying diffusion metrics calculated from tractography using the right PPN as the seed ROI for unrestricted **(A)** and targeted tractography **(B–F)**, FA **(B)**, MD **(C)**, RD **(D)**, AD **(E)**, and the tract volume **(F)**. *p < 0.05 comparing between the control and PD the group. AD, axial diffusivity; FA, fractional anisotropy; MD, mean diffusivity; RD, radial diffusivity; GPe, globus pallidus externa; GPi, globus pallidus interna; M1, primary motor cortex; PMd, dorsal premotor cortex; PMv, ventral premotor cortex; S1, primary sensory cortex; SMA, supplementary motor area; pre-SMA, pre-supplementary motor area; STN, subthalamic nucleus.

Probabilistic tractography was used to assess the structural connectivity of the PPN (Figure 2). Unrestricted connectivity maps using the PPN as seed ROIs recapitulated previously reported widespread connections of the PPN to the cerebral cortex, particularly the primary motor cortex, dorsal pre-motor cortex, supplementary and pre-supplementary motor areas, and to a lesser extent the somatosensory cortex and ventral premotor cortex. Superior projections to subcortical structures included the thalamus, subthalamic nucleus, substantia nigra, globus pallidus interna, dorsal striatum, and amygdala. Descending fiber tracts involved the cerebellum, primarily *via* the inferior cerebellar peduncle and corticopontine tracts, and targets within the spinal cord (Figure 2; Fling et al., 2013). No differences were observed between the connectivity maps of PD and control subjects by visual inspection. With respect to diffusion metrics, AD in the unrestricted right hemispheric tracts was significantly higher in PD versus control subjects (*p* = 0.038) but did not maintain significance following correction for false discovery; no statistically significant differences were observed in the left hemisphere (Figures 3A, 4A).

To determine if the integrity of specific PPN networks were altered in PD subjects, restricted tractography between the PPN and cortical or subcortical targets known to be critical for locomotor control and cognition was performed. No statistically significant differences in tract volume, FA, MD, RD, or AD were observed between PD and control subjects in the left hemisphere (Figure 3). By contrast, reduced structural integrity in PD subjects was observed in specific tracts within the right hemisphere (Figure 4): tract volume of PPN to the primary somatosensory cortex (S1; *p* = 0.028) and pre-supplementary motor area (pre-SMA; *p* = 0.012) was lower in the PD group; MD of PPN to caudate nucleus (*p* = 0.010) and amygdala (*p* = 0.004) were higher in the PD group; RD in PPN to caudate nucleus (*p* = 0.016) and amygdala (*p* = 0.016) were higher in the PD group; AD in PPN to caudate nucleus (*p* = 0.018) and amygdala (*p* = 0.001) were higher in the PD group. Following correction for repeated measures, both PPN-caudate nucleus and PPN-amygdala MD, RD, and AD values remained significant. Regarding PPN-STN connectivity, although there is a trend of increased MD and RD in the PD group in the right hemisphere, no statistical significance was observed (Figure 4). Localized abnormalities within a tract may not be apparent when averaging properties across the entire tract length, so along-the-tract analysis was added to spatially characterize diffusion abnormalities along the trajectory of each tract between PPN and target structure. When corrected for multiple comparisons, no statistically significant differences between PD and controls were observed (Supplementary Figure 2).

### Correlation Between Diffusion Metrics and Gait Parameters

**FIGURE 5:**
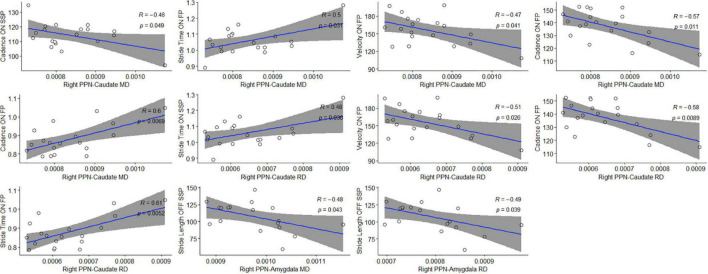
Statistically significant correlations between microstructural changes in locomotion centers and gait parameters. Pearson’s correlation coefficient (ρ) and significance values are shown for each plot; 95% confidence intervals are shaded in gray.

Correlation analysis was performed between diffusion metrics in the tracts of interest established by TBSS and targeted tractography versus gait assessments in both the ON and OFF states. In the case of TBSS-identified tracts of interest, no significant correlations were observed (Supplementary Table 1). With respect to the tracts of interest identified by targeted tractography, statistically significant correlations of moderate strength were observed between DTI measures indicative of reduced structural connectivity with measures of worsened gait (Table 5 and Figure 5). Specifically, higher MD values in the right PPN-caudate nucleus tracts were associated with reduced cadence (*r* = −0.457, *p* = 0.049) and higher MD and RD values were associated with increased stride time (*r* = 0.495, *p* = 0.032; and *r* = 0.484, *p* = 0.036) in the ON-state at SSP. Additionally, higher MD and higher RD in the right PPN-caudate nucleus tracts were both associated with reduced cadence (*r* = −0.571, *p* = 0.011; and *r* = −0.582, *p* = 0.009, respectively), reduced velocity (*r* = −0.472, *p* = 0.042; and *r* = −0.509, *p* = 0.027, respectively), and increased stride time (*r* = 0.597, *p* = 0.007; and *r* = 0.613, *p* = 0.006, respectively) in the ON-state at FP. Finally, both higher MD and RD in the right PPN-amygdala tracts were associated with reduced stride length (*r* = −0.481, *p* = 0.044; and *r* = −0.490 and *p* = 0.039, respectively) in the OFF state at SSP.

**TABLE 5:** Pearson’s correlation of microstructural changes in locomotion centers with gait parameters.

Gait parameters	State	Diffusion metrics (PPN to target tracts)					
		Right PPN-caudate			Right PPN-amygdala		
		MD	RD	AD	MD	RD	AD
Velocity SSP	ON	–0.38	–0.38	–0.29	0.1	0.05	0.19
	OFF	0.26	0.21	0.34	–0.39	–0.41	–0.32
Cadence SSP	ON	−0.46*	–0.44	–0.4	0.28	0.23	0.35
	OFF	0.04	0.03	0.05	0.11	0.14	0.06
Stride time SSP	ON	0.50*	0.48*	0.42	–0.3	–0.25	–0.39
	OFF	–0.04	–0.04	–0.05	–0.12	–0.14	–0.07
Stride length SSP	ON	–0.30	–0.31	–0.21	0.00	–0.04	0.09
	OFF	0.41	–0.04	0.44	−0.48*	−0.49*	–0.42
Velocity FP	ON	−0.47*	−0.51*	–0.28	0.03	–0.03	0.15
	OFF	0.04	–0.04	0.22	–0.4	–0.44	–0.27
Cadence FP	ON	−0.57*	−0.58**	–0.42	0.05	0.03	0.09
	OFF	–0.08	–0.15	0.14	–0.2	–0.21	–0.16
Stride time FP	ON	0.60**	0.61**	0.43	–0.09	–0.07	–0.13
	OFF	0.04	0.11	–0.17	0.2	0.21	0.16
Stride length FP	ON	–0.25	–0.29	–0.1	0.02	–0.04	0.14
	OFF	0.09	0.04	0.19	–0.37	–0.41	–0.25

Data are presented as Pearson’s correlation coefficient (r_s_) with statistically significant correlations (*p < 0.05, **p < 0.01) bolded. SSP, self-selected pace; FP, fast pace.

## Discussion

In this study, we investigated alterations of structural connectivity affecting PPN networks in PD using both whole-brain and targeted tractography analyses combined with detailed gait assessments in both the ON and OFF states focusing on the pace and rhythm domains of gait. The main findings, observed with probabilistic tractography, were (1) significant interruptions in PPN-caudate nucleus and PPN-amygdala connectivity pathways, which (2) were correlated with alterations in gait parameters. Specifically, disruption of the right PPN-caudate nucleus pathway was associated with worsened cadence, stride time, and velocity while in the ON state, whereas disruption in the right PPN-amygdala pathway was associated with reduced stride length in the OFF state. These results suggest that the strength of right hemispheric PPN-caudate nucleus and PPN-amygdala connections are associated with gait impairment in PD. Although altered connectivity between PD and controls was observed when applying TBSS, no statistically significant correlations with gait metrics were found.

The PPN is well-poised to act as a key modulator of locomotion, due to widespread connections with the brainstem, spinal cord, cerebellum, cortex, thalamus, and reciprocal connections with the basal ganglia and limbic system (French and Muthusamy, 2018). Previous studies have demonstrated a role for PPN dysfunction in PD, specifically in relation to balance and gait. Reduced connectivity of the PPN is associated with FOG (Fling et al., 2013, 2014), while reduced PPN structural integrity predicts the development of postural instability and gait difficulties (Craig et al., 2020). Although both cholinergic and non-cholinergic neurons comprise the PPN, evidence suggests that cholinergic dysfunction appears to contribute more to gait disturbance and postural instability. Gait and balance dysfunction tend to respond poorly to dopaminergic agents (Smulders et al., 2016) and extensive cholinergic loss in the PPN is observed in PD (Hirsch et al., 1987). Targeted lesions of cholinergic neurons in the PPN induce gait and postural deficits (Karachi et al., 2010), while reduced gait speed in PD is associated with reduced cholinergic activity and increased cholinergic degeneration (Rochester et al., 2012; Bohnen et al., 2013). On the other hand, the methodology used in this study is not able to determine which neurochemical projections of the PPN are affected (whether cholinergic or non-cholinergic). Although the cholinergic system is clearly involved in gait control, the PPN comprises a heterogenous cell population, and we cannot discount the involvement of GABAergic and glutamatergic signaling dysfunction in PD. More recent evidence has also shown a substantial loss of these neuronal populations in PD subjects, particularly those with falls (Sebille et al., 2019). Less is known about the connectivity patterns of non-cholinergic cells of the PPN, although there appears to be substantial overlap with cholinergic targets, including the substantia nigra, subthalamic nucleus, thalamus, and ventral tegmental area (Nowacki et al., 2019). The interplay between these signaling pathways appears to be more complex than originally devised, since stimulation of glutamatergic and GABAergic neuronal populations in experimental models can induce or stop locomotion, respectively, whereas cholinergic signaling appears to have a more modulating role (Roseberry et al., 2016), although such subpopulations may be differentially recruited depending on type of body movement (Ferreira-Pinto et al., 2021). Of note, when comparing gait parameters between PD subjects’ ON and OFF states, only non-significant trends were observed. This would be in keeping with the fact that gait is influenced by other (i.e., cholinergic) signaling.

This study showed that alterations of connectivity between the right PPN and caudate nucleus were moderately associated with worsened gait metrics only in the ON state, presumably when dopaminergic signaling is optimized. The PPN provides a significant source of direct cholinergic and glutamatergic innervation to the dorsal striatum (Dautan et al., 2014, 2021; Assous et al., 2019), and direct projections between the PPN and caudate nucleus have been described in animal models (Saper and Loewy, 1982; Nakano et al., 1990). Experimental lesions or stimulation of the PPN appears to influence striatal dopamine and acetylcholine activity (Jenkins et al., 2002; Forster and Blaha, 2003). We have previously shown that microstructural alterations in the caudate nucleus are correlated with gait abnormalities (Surkont et al., 2021), and a relationship between reduced dopaminergic function in the caudate nucleus and the development of postural and gait impairment has been demonstrated by others (Kim et al., 2018, 2019; Craig et al., 2020). Similar to the association between reduced caudate innervation and metrics related to gait speed (i.e., cadence, stride time, and overall gait velocity) observed in this study, gait velocity was correlated with caudate atrophy in subjects with mild cognitive impairment (Beauchet et al., 2019). More specific evidence of a direct relationship between direct PPN-striatal signaling comes from the observation that selective activation of PPN cholinergic neurons can restore axial-related motor dysfunction by restoring nigral and striatal dopamine release (Sharma et al., 2020), whereas glutamatergic excitatory input from the PPN suppresses striatal output by feedforward inhibition (Assous et al., 2019). Going further, a role for the importance of PPN-caudate connections with mobility is suggested from imaging studies which demonstrated reduced cholinergic innervation of the right caudate nucleus in PD subjects with FOG (Bohnen et al., 2019). It is therefore reasonable to speculate that failure in both dopaminergic and non-dopaminergic-dependent signaling between the right PPN and caudate nucleus may additionally underlie gait abnormalities in PD.

We observed that different DTI parameters of the right PPN-caudate tract correlate differently with specific gait parameters, for example, MD/RD showed significant correlations, but AD/FA did not. Also, there were differences in correlation between gait parameters at SSP versus FP, i.e., MD/RD of the right PPN-caudate showed a correlation with velocity FP, but not with velocity SSP. The velocity FP has been proposed to be an indicator of gait velocity reserve and the change predicts disability (Artaud et al., 2015). Although caution is required, the correlation between MD/RD, but not AD/FA, and the difference in FP versus SSP raises the possibility that specific aspects of tract morphology might contribute to the relationship with gait parameters.

Beyond its proposed functions as an integrator of sensorimotor information, the PPN additionally serves functions related to cognition, arousal, and emotional information (French and Muthusamy, 2018). The PPN is known to project to the central nucleus of the amygdala and receive inputs from the limbic cortex (Sebille et al., 2017). In this study, we observed altered connectivity between the right PPN and amygdala. The central nucleus of the amygdala has previously been implicated in mediating fear- and anxiety-related freezing behavior in animal models (Kalin et al., 2004), which may resemble the FOG observed in PD. In addition, anxiety can independently contribute to the severity of FOG (Pimenta et al., 2019), and in PD patients with gait impairment or FOG, cognitively- or sensorily challenging situations are thought to cause an over-activation of striatal output nuclei (Lewis and Shine, 2016). Such over-activation may lead to enhanced GABAergic inhibitory projections to thalamic and brainstem locomotor regions (Lewis and Shine, 2016). Thus, psychophysiological responses of anxiety, fear of falling, and autonomic changes occurring during FOG (Maidan et al., 2010; Mazilu et al., 2015) suggest the involvement of the amygdala in the manifestation of FOG. In keeping with this, PD patients with a PIGD subtype have greater amygdalar gray matter loss compared to tremor-dominant patients and age-matched healthy controls (Burn et al., 2012; Rosenberg-Katz et al., 2016). One could thus speculate a link between the PPN and cortical-subcortical networks linked to fear and arousal leading to FOG and gait instability. Some experimental evidence supports this, since glutamatergic neurons in the PPN receive prominent innervation from the amygdaloid complex, along with the basal ganglia, and laterodorsal tegmental nucleus, and their stimulation can elicit locomotion (Roseberry et al., 2016). Since our patient group were PD patients with gait impairment, the change in the amygdala can be a mechanism to explain the correlation between the MD and RD in the right PPN-amygdala tracts with the changes in stride length, which is commonly seen in FOG.

Whole brain connectivity analysis using TBSS demonstrated dysfunctional network connectivity involving frontal circuits and the corpus callosum, which is consistent with previous studies (Hall et al., 2018; Li et al., 2018; Salsone et al., 2019; Wei et al., 2020). However, no significant correlations between these networks with gait parameters were observed in this study. The corpus callosum is heavily involved in communicating a wealth of sensory, motor, and cognitive information between the two hemispheres. Therefore, although a clear relationship between reduced corpus callosal integrity and PD is consistently seen, it is likely not exclusive to gait abnormalities alone, and thus probably has less discriminatory power. TBSS is also limited by its inability to examine specific tracts such as PPN connections.

The tractography analysis identified significant structural connectivity changes in the right hemisphere only, consistent with previous reports. Lateralization of the structural and functional connectivity in the human brain has been reported in multiple studies involving people with gait and postural impairment – specifically, FOG – and was strongly related to structural deficits in the right hemisphere’s locomotor network (Bartels et al., 2006; Bartels and Leenders, 2008; Snijders et al., 2011; Fling et al., 2013). The present data does not allow for specific comments regarding the substrate of FOG since most participants had FOG and we did not include a non-FOG comparative group. Apart from FOG, right hemispheric networks may be preferentially involved in modulating gait, since key functions, including visuospatial orientation, proprioceptive processing, and cognitive inhibitory control, are lateralized to the right hemisphere (Goble et al., 2012; Xu et al., 2017). In keeping with this, left-sided PD symptom onset (implicating predominant right-sided nigrostriatal dysfunction) is associated with increased risk of gait instability (Giladi et al., 2001), and right parietal cortex dysfunction is related to the severity of gait disturbance (Cremers et al., 2012). Thus, PPN network structural abnormalities restricted to the right hemisphere may have practical implications for deep brain stimulation (DBS). For example, unilateral left PPN stimulation in a small cohort improved axial symptoms at 12 months as compared to right PPN stimulation (Lam et al., 2015). This may have resulted from reduced white matter connectivity of the right PPN, in which DBS stimulation could be less effectively transmitted. Taken together, alterations in structural and functional connectivity involving the right hemisphere appear to be associated with impaired gait and balance, in support of our findings.

Strengths of this study include the use of quantitative gait measures in both the subjects’ ON and OFF states, which allow for precise evaluation of gait during optimized and unoptimized dopaminergic signaling, respectively. Another strength is the novelty in our analysis since there is poor evidence on the correlations between imaging and objective gait parameters in PD.

Limitations of this study include the fact that this a single-centered study with relatively small sample size, although, our participants are representative of the spectrum of gait impaired patients with PD. Sample size estimation assuming a correlation of 0.5 (i.e., moderate strength), significance level of 0.05, and 80% statistical power suggests a required cohort size of approximately 29 (R Core Team, 2021); therefore, this study would be underpowered, but serves as a pilot study for exploratory analysis. For example, although the PPN appears to strongly influence STN and motor cortex activity (Breit et al., 2006; Aravamuthan et al., 2008), only a non-significant trend in reduced PPN-STN and PPN-M1 connectivity was observed; with increased sample size in future studies, such differences may be found. Recent work in fact argues for the use of thousands of scans in order to produce reproducible brain-wide association studies (Marek et al., 2022), and thus, more definitive studies may require the leveraging of large databases, such as the Parkinson’s Progression Markers Initiative or Canadian Consortium for Neurodegeneration in Aging. Nonetheless, our data are consistent with the literature and go beyond the literature by examining both TBSS and tracts of interest. We show that TBSS changes do not reflect gait change in our population, supporting the specificity of our PPN tractography findings. With respect to methodology, although direct connections from the PPN to the sub-cortical and cortical targets have been demonstrated, at least in experimental models, probabilistic tractography includes both direct and indirect white matter connections, and therefore we cannot definitively establish that the pathways observed reflect a direct signaling pathway. In addition, descending projections from the PPN are thought to modulate locomotor centers in the spinal cord (French and Muthusamy, 2018); these important connections were not investigated, but can be explored in a future study.

Modulating PPN activity using DBS can sometimes demonstrate significant, though often modest and inconsistent, improvements in gait, balance, and FOG (Nowacki et al., 2019). This is certainly in part due to the great variability in targeting, stimulation, and outcome measurements among published studies. However, it also reflects the functional diversity of the PPN, which together with the cuneiform nucleus and mesencephalic reticular formation form the mesencephalic locomotor region (MLR). Recent studies have demonstrated that stimulation of discrete subpopulations of the MLR can activate, modulate, or inhibit locomotion (Dautan et al., 2021; Ferreira-Pinto et al., 2021). Moreover, such dedicated neuronal subpopulations appear to be recruited during different forms of body movement aside from locomotion. This intermingling of neurons of distinct functions within the MLR likely accounts for the difficulty in reliably targeting a complex motor symptom with PPN-DBS intervention, and indeed, many DBS groups have redirected efforts away from the PPN to focus upon other sub-targets within the MLR, such as cuneiform nucleus (Chang et al., 2020). For example, a recent study of MLR DBS showed that PD patients had a good response to stimulation when they had active electrode contacts either in or bordering the cuneiform nucleus. DBS in this area can significantly alleviate FOG (Goetz et al., 2019). Thus, teasing out the function of the other components of the MLR will be the focus of future studies.

## Conclusion

Our exploratory analysis detects a potential correlation between gait dysfunction in PD and a characteristic pattern of connectivity deficits in the PPN network involving the right caudate nucleus and right amygdala, which may be investigated in future, larger studies. The PPN DTI changes may represent subcortical locomotor network failure. Overall, DTI analyses might be a useful tool for assessing PD in specific motor domains, such as gait, and potentially could serve as an imaging biomarker.

## Data Availability Statement

There are restrictions to re-access to some clinical data. Requests to access these datasets should be directed to FB, fb@ualberta.ca.

## Ethics Statement

The studies involving human participants were reviewed and approved by the University of Alberta Health Research Ethics Board. The patients/participants provided their written informed consent to participate in this study.

## Author Contributions

SJ executed the research project, designed and executed the statistical analysis, and contributed to writing first draft of the manuscript. RC conceived the research project, designed, and reviewed and critiqued the statistical analysis and manuscript. WM conceived and organized the research project, and reviewed and critiqued the manuscript. MW conceived, organized, and executed the research project, and reviewed and critiqued the manuscript. MG executed the research project and, reviewed and critiqued the manuscript. FB conceived and organized the research project, designed, and reviewed and critiqued the statistical analysis and manuscript. All authors have approved the final article.

## Conflict of Interest

The authors declare that the research was conducted in the absence of any commercial or financial relationships that could be construed as a potential conflict of interest.

## Publisher’s Note

All claims expressed in this article are solely those of the authors and do not necessarily represent those of their affiliated organizations, or those of the publisher, the editors and the reviewers. Any product that may be evaluated in this article, or claim that may be made by its manufacturer, is not guaranteed or endorsed by the publisher.
