# Supplementary material for: Pedunculopontine Nucleus Dysconnectivity Correlates With Gait Impairment in Parkinson’s Disease: An Exploratory Study
Source: Front Aging Neurosci. 2022 Jul 8;14:874692. doi: 10.3389/fnagi.2022.874692 (PMC9304714; doi:10.3389/fnagi.2022.874692)
Supplement: Supplementary file 1 [file Data_Sheet_1.docx]

Supplementary Materials

**Supplementary Figure S1**


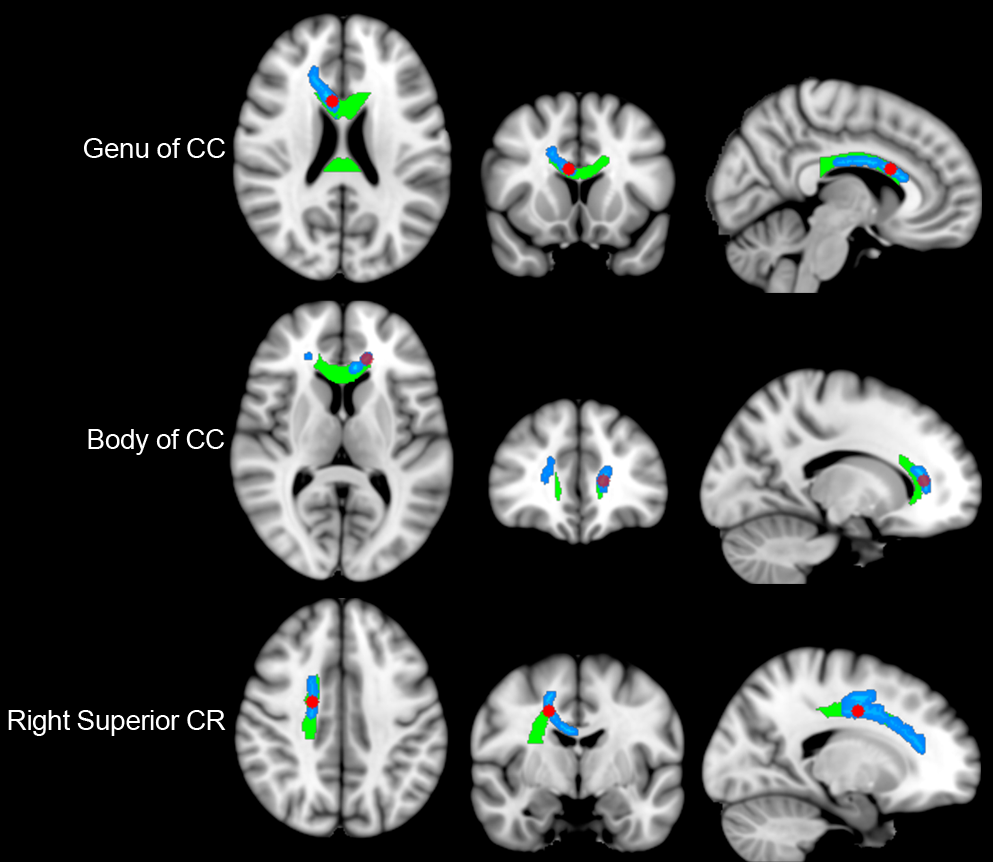


Areas of overlap between clusters of reduced FA in PD relative to control subjects as determined by TBSS (blue) and white matter tracts derived from the JHU ICBM-DTI-81 White Matter Labels Atlas (green), including the genu and body of the corpus callosum and the right superior corona radiata. Spheres of 4 mm diameter with center at peak coordinates in each cluster are also shown (red). Axial and coronal sections are orientated by radiological convention (left hemisphere is on the right side). CC: corpus callosum, CR: corona radiata.

**Supplementary Figure S2: Along-the-tract analyses of diffusion metrics between PPN seed and target.**


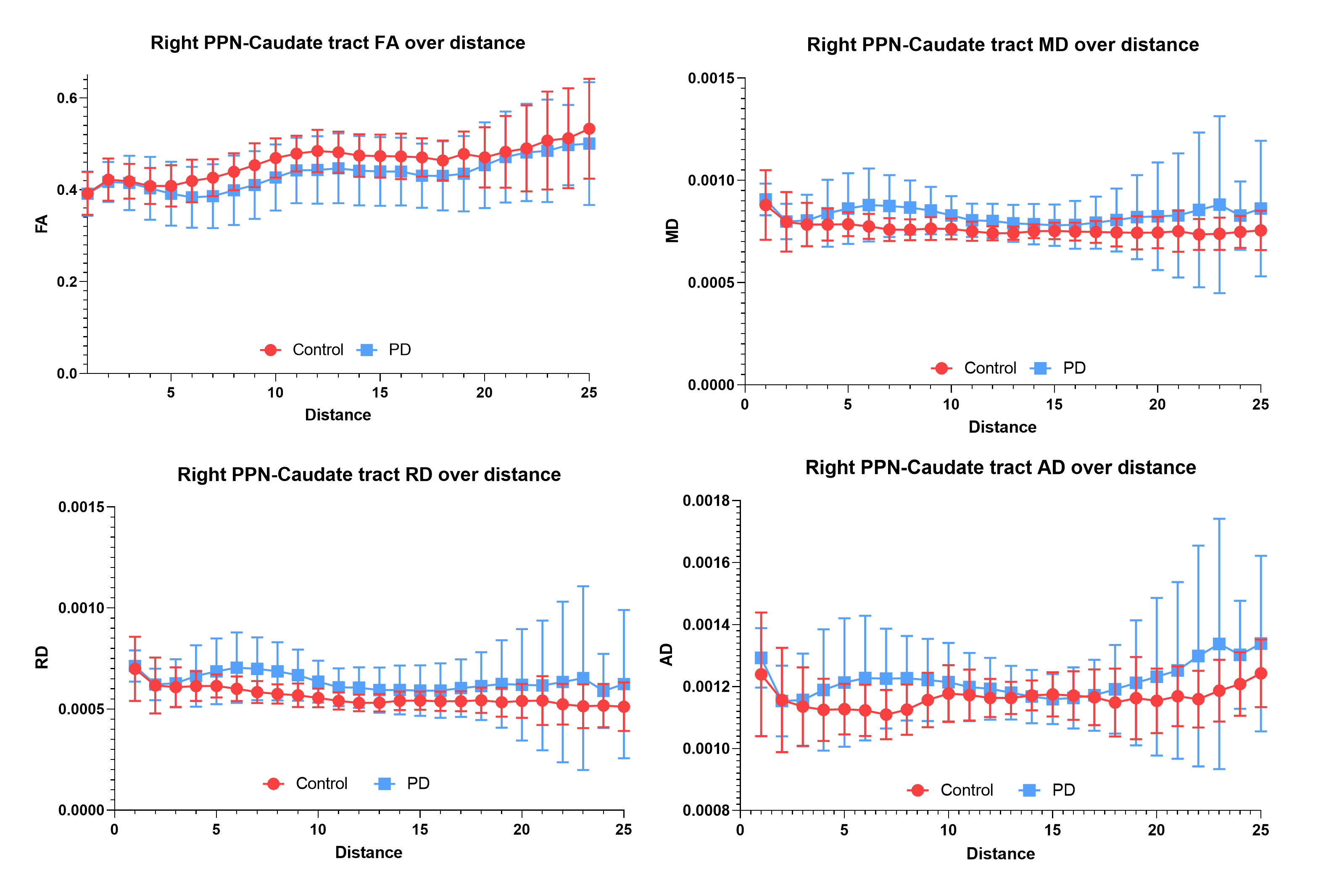


Analyses between the Right PPN and caudate nucleus are shown for illustrative purposes; additional analyses were conducted for all 28 PPN-to-target tracts. After correction for multiple comparisons, no statistically significant differences were identified

**Supplementary Table S1: Pearson’s correlation of mean FA changes in white matter areas of interest identified by TBSS in PD versus control subjects with gait parameters**

| **Gait Parameters** | **State** | **Diffusion metrics** | | | | | |
| --- | --- | --- | --- | --- | --- | --- | --- |
|  |  | **Body of CC** | | **Genu of CC** | | **Right Superior CR** | |
|  |  | **Whole tract** | **Sphere** | **Whole tract** | **Sphere** | **Whole tract** | **Sphere** |
| **Velocity SSP** | ON | 0.015/0.954 | -0.234/0.337 | 0.212/0.385 | 0.229/0.348 | 0.17/0.489 | 0.365/0.125 |
|  | OFF | 0.095/0.702 | -0.002/0.994 | 0.021/0.933 | 0.129/0.601 | 0.329/0.17 | -0.019/0.939 |
| **Cadence SSP** | ON | 0.002/0.996 | -0.144/0.559 | 0.134/0.587 | 0.18/0.461 | -0.063/0.799 | 0.138/0.576 |
|  | OFF | 0.227/0.366 | -0.124/0.625 | 0.396/0.105 | 0.201/0.426 | 0.08/0.754 | -0.115/0.651 |
| **Stride Time SSP** | ON | 0.005/0.984 | 0.184/0.453 | -0.09/0.717 | -0.162/0.508 | 0.055/0.826 | -0.153/0.533 |
|  | OFF | -0.23/0.361 | 0.117/0.645 | -0.384/0.117 | -0.202/0.423 | -0.093/0.717 | 0.118/0.644 |
| **Stride Length SSP** | ON | -0.014/0.956 | -0.241/0.322 | 0.166/0.499 | 0.166/0.5 | 0.211/0.387 | 0.386/0.103 |
|  | OFF | 0.022/0.934 | 0.061/0.813 | -0.081/0.752 | 0.068/0.792 | 0.317/0.201 | 0.002/0.997 |
| **Velocity FP** | ON | 0.018/0.942 | -0.335/0.162 | 0.288/0.233 | 0.214/0.381 | 0.292/0.226 | 0.247/0.31 |
|  | OFF | 0.025/0.921 | -0.158/0.521 | -0.038/0.878 | 0.044/0.86 | 0.254/0.296 | -0.05/0.841 |
| **Cadence FP** | ON | 0.039/0.875 | -0.286/0.236 | 0.111/0.653 | 0.288/0.233 | 0.113/0.648 | -0.203/0.406 |
|  | OFF | 0.152/0.537 | -0.372/0.118 | -0.01/0.968 | 0.01/0.968 | 0.018/0.944 | -0.368/0.122 |
| **Stride Time FP** | ON | -0.003/0.992 | 0.291/0.228 | -0.127/0.607 | -0.286/0.236 | -0.12/0.628 | 0.175/0.476 |
|  | OFF | -0.163/0.506 | 0.327/0.172 | 0.004/0.988 | -0.052/0.835 | -0.088/0.722 | 0.341/0.154 |
| **Stride Length FP** | ON | -0.026/0.919 | -0.259/0.286 | 0.25/0.304 | 0.06/0.808 | 0.247/0.308 | 0.413/0.079 |
|  | OFF | -0.077/0.756 | 0.017/0.948 | -0.037/0.882 | 0.04/0.872 | 0.289/0.232 | 0.138/0.576 |

Data are presented as Pearson’s correlation coefficient (r_s_) / p-value. No statistically significant correlations were observed. CC: corpus callosum, CR: corona radiata, SSP: self-selected pace, FP: fast pace
